# Optimizing the synergy between stereotactic radiosurgery and immunotherapy for brain metastases

**DOI:** 10.3389/fonc.2023.1223599

**Published:** 2023-08-11

**Authors:** Kelly H. Yoo, David J. Park, John H. Choi, Neelan J. Marianayagam, Michael Lim, Antonio Meola, Steven D. Chang

**Affiliations:** Department of Neurosurgery, Stanford University School of Medicine, Stanford, CA, United States

**Keywords:** stereotactic radiosurgery, immunotherapy, immune checkpoint inhibitor, brain metastasis, combination therapy

## Abstract

Solid tumors metastasizing to the brain are a frequent occurrence with an estimated incidence of approximately 30% of all cases. The longstanding conventional standard of care comprises surgical resection and whole-brain radiotherapy (WBRT); however, this approach is associated with limited long-term survival and local control outcomes. Consequently, stereotactic radiosurgery (SRS) has emerged as a potential alternative approach. The primary aim of SRS has been to improve long-term control rates. Nevertheless, rare observations of abscopal or out-of-field effects have sparked interest in the potential to elicit antitumor immunity *via* the administration of high-dose radiation. The blood-brain barrier (BBB) has traditionally posed a significant challenge to the efficacy of systemic therapy in managing intracranial metastasis. However, recent insights into the immune-brain interface and the development of immunotherapeutic agents have shown promise in preclinical and early-phase clinical trials. Researchers have investigated combining immunotherapy with SRS to enhance treatment outcomes in patients with brain metastasis. The combination approach aims to optimize long-term control and overall survival (OS) outcomes by leveraging the synergistic effects of both therapies. Initial findings have been encouraging in the management of various intracranial metastases, while further studies are required to determine the optimal order of administration, radiation doses, and fractionation regimens that have the potential for the best tumor response. Currently, several clinical trials are underway to assess the safety and efficacy of administering immunotherapeutic agents concurrently or consecutively with SRS. In this review, we conduct a comprehensive analysis of the advantages and drawbacks of integrating immunotherapy into conventional SRS protocols for the treatment of intracranial metastasis.

## Introduction

Brain metastasis describes the dissemination of neoplastic cells from a primary malignancy to the brain tissue and is a common complication in adults with solid tumors (1, 2). The incidence rates vary and are commonly observed in patients with lung, melanoma, renal cell, and breast cancers (3). Various treatment modalities are available to manage brain metastasis, including chemotherapy (CT), surgical intervention, whole-brain radiotherapy (WBRT), stereotactic radiosurgery (SRS), targeted therapies, and immunotherapy (4–6).

SRS has emerged as a popular choice among these modalities, primarily due to its superior efficacy and reduced toxicity compared to WBRT (7). WBRT triggers double-stranded DNA damage, leading to the generation of cytotoxic free radicals in the tumor cells due to oxygenation (8). In contrast, high-precision SRS elicits a local and systemic immune response against cancerous cells, resulting in better long-term control rates and a lower risk of neurocognitive decline when compared to conventional WBRT (9).

Animal studies have recently shown the occurrence of the abscopal effect (AE), a phenomenon in which the combination of radiation and dendritic cell growth factor leads to a reduction in distant metastases and improved disease-free survival compared to radiation alone (10). This effect occurs due to the ability to activate an immune response against cancer cells. The incorporation of immunotherapeutic agents that enhance the host immune response against cancer has expanded the range of therapeutic options available for neoplastic diseases (11). Anti–cytotoxic T-lymphocyte–associated antigen 4 (Anti-CTLA-4) and anti–programmed death 1/programmed death ligand 1 (anti-PD-1/PD-L1) antibodies have emerged as a key component of treatment for a range of tumors. However, the optimal combination and timing of these systemic agents with radiation therapy (RT) remains to be fully elucidated (12).

Recent advances in immunotherapy have led to the re-evaluation of the potential impact of RT, particularly through the use of hypo-fractionated ablative irradiation (13). The mechanism linking radiation dose and fractionation to antitumor immunity holds substantial implications for clinical translation and the potential synergistic effects with immunotherapy through the release of tumor-associated antigens (TAA), improved antigen presentation, and increased infiltration of immune cells into the tumor microenvironment (TME) (14). However, further investigation is crucial to optimize the combination of SRS and novel immunotherapies.

## Methods

A systematic literature search was performed in the PubMed database, limited to articles published in the English language through April 28, 2023. The search strategy employed different keywords related to immunotherapy for the treatment of intracranial melanoma (M), non-small cell lung carcinoma (NSCLC), renal cell carcinoma (RCC), and other relevant malignancies. The identified publications were subjected to a screening process, during which non-English articles, as well as publications that fell under the categories of reviews, editorials, commentaries, case reports, opinion letters, and viewpoints, were excluded.

**Figure 1:**
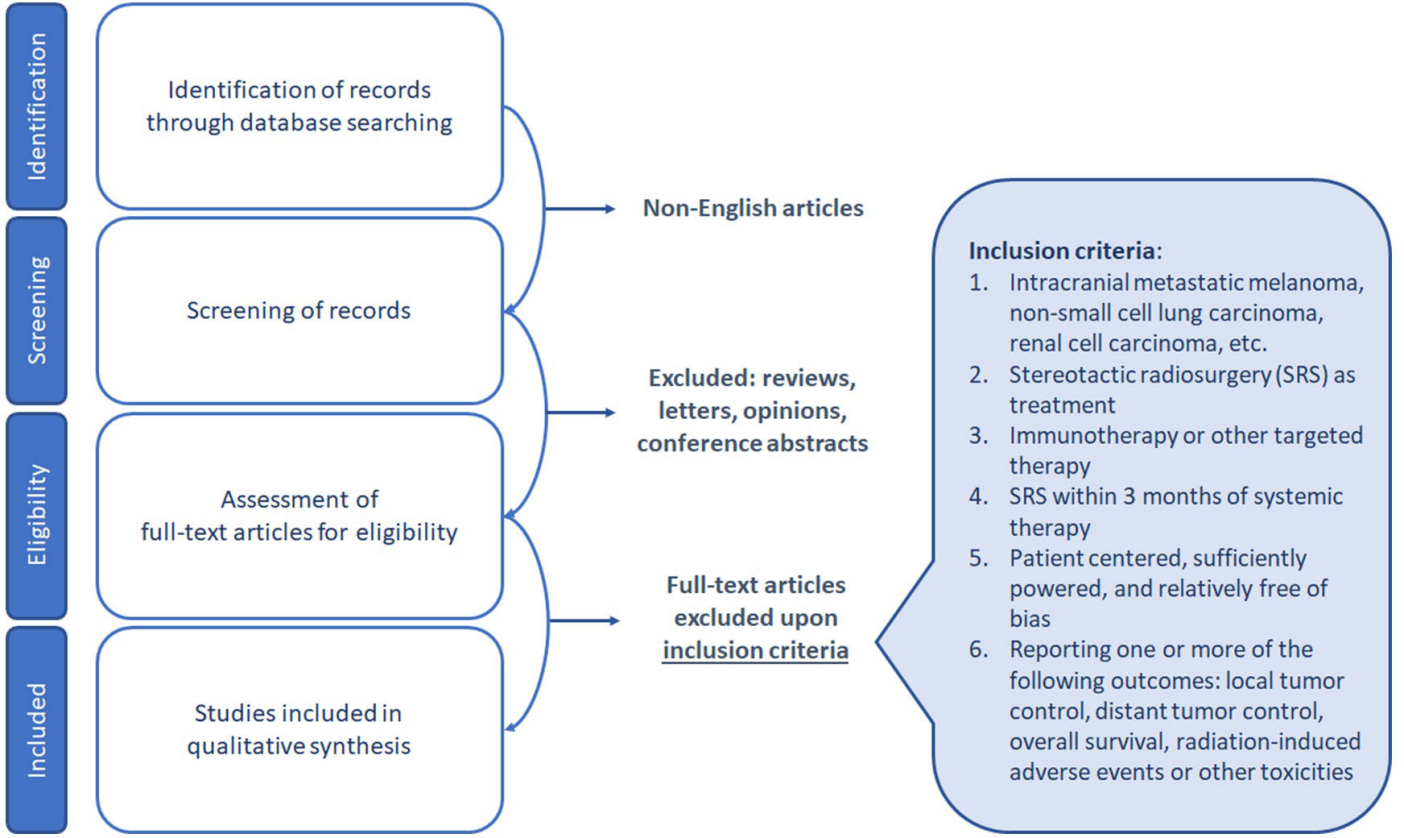
Diagram of PRISMA workflow with search strategy and inclusion criteria.

The eligibility criteria for inclusion in the qualitative synthesis were as follows: patient-centered studies with SRS as a treatment modality within three months of systemic therapy, and relatively unbiased reporting of one or more of the following outcomes: local tumor control, distant tumor control, overall survival, radiation-induced adverse events, or other toxicities. The selection of studies for qualitative synthesis was based on a rigorous evaluation of the study design, patient population, and quality of reporting (**Figure 1**).

## Role of stereotactic radiosurgery in management of brain metastases

The therapeutic landscape for brain metastases has undergone significant evolution in recent years, with SRS emerging as the preferred treatment modality for patients with multiple intracranial metastases (15). The preference for SRS as a treatment option for multiple intracranial metastases has stemmed from the recognition that WBRT does not confer significant survival benefits and may adversely impact neurocognitive function, in contrast to SRS (9). SRS has consistently demonstrated high long-term control rates, with estimates of at least 70% at one year for SRS alone, and even higher rates for smaller metastases. Despite the favorable outcomes of SRS in treating brain metastases, studies estimate that a considerable proportion of patients (30-50%) may develop new distant brain metastases over the same period. As a strategy to minimize the risks of radiation-related toxicities and costs, and to defer or avoid the use of WBRT and its associated adverse effects, many patients undergo multiple rounds of SRS before considering the option of WBRT if necessary (16).

## Immuno-modulation by stereotactic radiosurgery

The process of stimulating the area undergoing SRS results in a complex set of physiological responses that can be broadly classified into two categories: (1) at the level of tumor cells, where it induces significant DNA damage leading to cell death, and (2) at the level of the TME, where it activates multiple signaling pathways, inducing a pro-inflammatory state within the TME, and potentially causing harm to the surrounding stromal and endothelial cells (**Table 1**) (17).

**Table 1:** Mechanism of action of immunomodulation by stereotactic radiosurgery.

Sequence of Action	Mechanism of SRS
1. Activation of DCs by induction of immunogenic cell death	Induction of STING pathway and type 1 IFN
2. Upregulation of CD8+ T cells by increased TAA presentation	Increase of expression of surface molecules (Fas, MHC class I, ICAM-1, CEA, or mucin)
3. Immunomodulation of TME	a. Induction of local production of chemokines, cytokines, and other soluble factors b. Alterations in tumor-associated stroma and endothelium c. Modulation of immune cell subsets in TME

SRS, stereotactic radiosurgery; DC, dendritic cell; IFN, interferon; STING, stimulator of interferon genes; MHC, major histocompatibility complex; ICAM, intracellular adhesion molecule; CEA, carcinoembryonic antigen; TME, tumor microenvironment.

**Figure 2:**
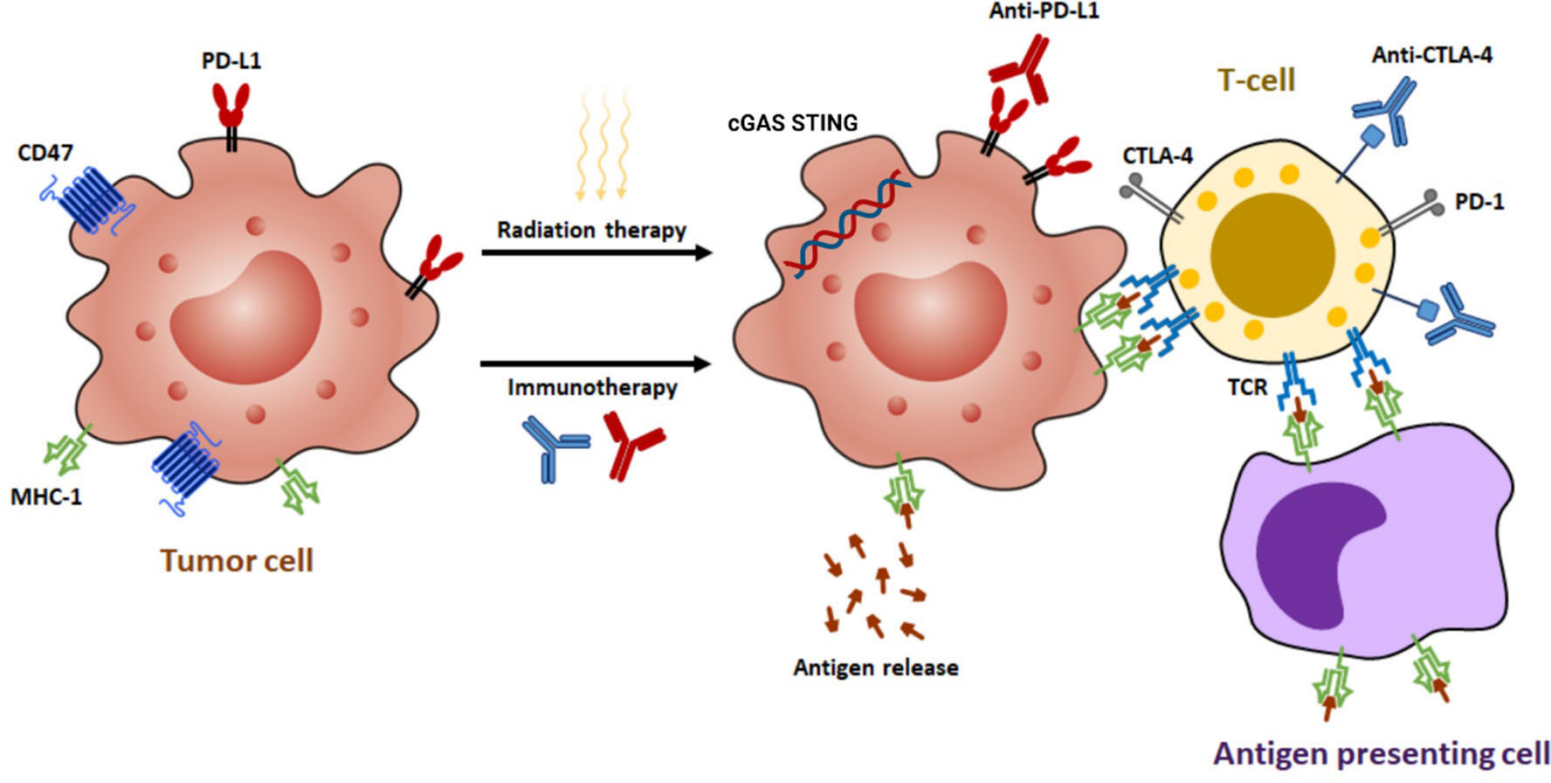
The synergistic effects of radiotherapy and immunotherapy mediated by various mechanisms. Radiation enhances the ability of antigen-presenting cells to present tumor antigens to naive T cells through the release of antigens, the stimulation of calreticulin, and the downregulation of CD47. This process leads to the expression of major histocompatibility complex class I (MHC-I) molecules and subsequent antigen presentation, which in turn results in the interaction between T-cell receptors (TCRs) and antigens. Moderate doses of radiation activate a type I interferon response by sensing cytoplasmic DNA *via* cyclic GMP-AMP synthase (cGAS)-stimulator of interferon genes (STING) pathway. In addition, radiation can upregulate programmed death-ligand 1 (PD-L1) and cytotoxic T-lymphocyte-associated protein 4 (CTLA-4), making immunotherapy a potential strategy to augment radiation efficacy by targeting these pathways.

The interaction between radiation and the host’s immune response to brain metastases is multifaceted and influenced by numerous factors. Brain metastases can escape immune detection through several mechanisms, including the secretion of cytokines that suppress immune activity, decreased expression of TAA and major histocompatibility complex (MHC) class I, and the recruitment of regulatory T cells (Tregs) to the TME (**Figure 2**) (18). In the vicinity of the tumor, Tregs can increase in proportion to as high as 20-30% of CD4+ T cells. Furthermore, the suboptimal functioning of host dendritic cells (DCs) also contributes to the weakened immune response to tumor cells, even in the presence of radiation (19).

Radiation has been shown to augment the presentation of TAA by DCs to both CD4+ and CD8+ T cells, thereby reinforcing the ability of the immune system to recognize and target tumor cells (20). Furthermore, radiation has been observed to facilitate the maturation of antigen-presenting cells (APCs), enhance the assembly of antigen-MHC complexes, and induce the secretion of critical inflammatory cytokines, including tumor necrosis factor-alpha (TNF-α), interferon gamma (IFN-β), and chemokine ligand 16 (CXCL16). These cytokines were found to attract immune cells to cross the blood-brain barrier (BBB) and infiltrate the TME (13).

In mouse models, single-fraction doses of 15 to 25 Gy have been demonstrated to elicit a CD8+ T-cell dependent immune response, leading to regression of the treated tumor. Depletion of CD8+ T cells has been associated with local tumor persistence, increased distant metastases, and decreased survival (21). Combining extracranial SRS with anti-PD-1 therapy has been shown to enhance the ratio of antigen-specific effector T cells to Tregs and increase T-cell infiltration into tumors, when compared to single-modality treatments, according to a study by Sharabi et al. (14).

## Current insights into the immune response of the central nervous system

Recent discoveries have challenged the conventional idea that the brain is an “immunologically privileged” site due to the BBB and the absence of lymphatic drainage. Radiolabeled antigens have been found to travel through the subarachnoid space to cervical and retropharyngeal lymph nodes (22), and dendritic cells have been identified in the meninges and choroid plexus, involved in antigen presentation to T cells (23). The BBB can also be affected by brain cancer and RT, leading to increased permeability and lymphocyte accessibility (24).

Studies have shown that radiation exposure can increase MHC class I expression on glioma cells, leading to the infiltration of CD4+ and CD8+ T cells, while systemic administration of anti-CTLA-4 antibodies has been found to enhance the effector T cell response and decrease the number of Tregs (25). Combining SRS and immunotherapy has shown efficacy in the management of brain metastases, leading to an improved local tumor response, deferred progression, reduction in size of unirradiated brain lesions, and prevention of new brain and systemic metastases through the AE (26–28).

The AE is defined as the suppression of unirradiated distant tumors or metastases following RT to a target lesion. The underlying mechanism is thought to be immune-mediated, where RT activates the immune system by revealing tumor-specific antigens that are processed by dendritic cells to activate T cells in neighboring lymph nodes (29). Research into the AE is ongoing, and there is growing interest in exploring its potential as a treatment strategy, particularly with the use of immune checkpoint inhibitors (ICI) which display a higher degree of immunomodulatory activity compared to other therapeutic approaches. However, AE remains a topic of significant controversy within the field of RT. To date, only one study has reported a case of AE in SRS (30).

## Immune checkpoint inhibitors

The human immune system plays a crucial role in defending against cancerous cells (31). Among the immunocompetent cells, T-cells have been identified as the most important ones in generating an antitumoral immune response (32). The potency of this response is determined by the modulation of stimulatory and inhibitory signals. The two immune checkpoints, CTLA-4 and PD-1, are crucial in regulating this response (33, 34). Antibodies such as ipilimumab, which targets CTLA-4, and nivolumab and pembrolizumab, which target PD-1, have shown promising results in cancer immunotherapy (**Figure 2**). These antibodies obstruct the inhibitory signals, amplifying the T-cell mediated immune response against cancer, and have been effectively used to manage various cancers (35).

## Interactions between radiotherapy and the immune system

SRS is a highly effective method for treating tumors and achieving improved local tumor control (LTC). However, distant relapse remains a common issue after exclusive SRS due to the persistence of an immunosuppressive TME. To address this issue, the incorporation of immune evasion inhibitors alongside SRS may be beneficial, potentially enhancing the antitumor immune response and overall treatment outcomes.

The fundamental impact of ionizing radiation on biological systems is mainly attributed to the damage it inflicts on DNA molecules. Radiation’s immune-stimulating effects have been extensively studied over the past two decades. Studies have shown that local RT can enhance the systemic immune response by releasing TAAs from necrotic and apoptotic tumor cell debris. These antigens are then presented to CD8+ cytotoxic T cells by DCs, initiating an immune response that attacks tumor cells in other parts of the body where the antigens are recognized (36). Preclinical and clinical studies have further demonstrated that combining therapeutic radiation with ICIs can significantly enhance the systemic immune response, resulting in immunogenic tumor cell death (37, 38).

The optimal timing and dosage of radiation to maximize antitumoral immune stimulation have been elucidated through several research studies. For instance, Schaue et al. conducted a study on a mouse M model to examine the effects of total dose, dose per fraction, and number of fractions of RT on the RT-induced immune response and the outcomes (39). Tumor growth was effectively inhibited by single fraction doses of radiation. The LTC rates were positively correlated with radiation dose and quantity of tumor-reactive T cells (21).

The parallel between the sequence of SRS and ICIs and the potential benefits observed with neoadjuvant, concurrent, and adjuvant ICI utilization offers a valuable perspective for optimizing the administration of SRS and ICIs in the context of brain metastases (40). The timing of ICI administration emerges as a critical determinant of therapeutic outcomes. Studies have shown that administering ICI therapy 2 to 4 weeks before initiating SRS treatment during the first cycle yields the most favorable results in terms of long-term control and overall survival (OS). Concurrent use of both therapies demonstrated the highest effectiveness (41, 42). Nevertheless, the efficacy of different ICIs may be influenced by their specific sequence and timing in relation to SRS treatment (41).

Administering ICIs prior to SRS holds promise in priming the immune system and enhance its response to SRS, resulting in improved LTC while inducing systemic antitumor effects (43). Notably, encouraging results have been demonstrated in M patients receiving neoadjuvant ICI therapy, particularly when combined with agents like pembrolizumab or nivolumab in conjunction with SRS or local therapies. Nonetheless, the efficacy and safety of the neoadjuvant approach require further investigation, especially in the context of brain metastases (44). The adjuvant utilization of ICIs has demonstrated significant potential in reducing the risk of cancer recurrence and metastasis. Remarkably, adjuvant ICI treatment following SRS has led to improved progression-free survival (PFS) and OS in high-risk M patients (45).

The determination of the optimal sequencing and timing of SRS and ICIs remains an active area of investigation through ongoing research and clinical trials. The effectiveness and safety of these combined treatment approaches may vary based on factors such as tumor type, patient characteristics, and treatment regimens (46). As the field of oncology continues to evolve, we anticipate that further data and evidence will emerge regarding the neoadjuvant and adjuvant administration of ICIs in combination with SRS across different cancer types. To inform treatment decisions effectively, it is of utmost importance to stay up-to-date with the latest literature and clinical trial results (**Tables 2**, **3**).

**Table 2:** Clinical trials of combination therapy in NSCLC, M, RCC, and other patients with brain metastasis.

Authors (year)			# Patients	Primary	Design	ICI	LTC (%)	Median OS (mo)
NSCLC								
SRS/ICI mono	Lee et al. (2021)	(47)	26 24 27	NSCLC	ICI mono Concurrent Non-concurrent	Nivolumab or pembrolizumab	NA	10.0 22.5 42.1
	Enright et al. (2020)	(48)	33 44	NSCLC	SRS + ICI SRS mono	Nivolumab or pembrolizumab or atezolimumab	97 86	13.9
	Shepard et al. (2019)	(49)	34 17	NSCLC	ICI mono SRS + ICI	Nivolumab or pembrolizumab or atezolimumab	76.3 84.9	15.9 NA
Concurrent	Lee et al. (2021)	(47)	26 24 27	NSCLC	ICI mono Concurrent Non-concurrent	Nivolumab or pembrolizumab	NA	10.0 22.5 42.1
	Schapira et al. (2018)	(50)	8 29	NSCLC	Concurrent Non-concurrent	Nivolumab or pembrolizumab or atezolimumab	100 77	17.6
SRS + ICI	Sign.C et al. (2020)	(51)	46 39	NSCLC	SRS + CT SRS + ICI	Nivolumab or pembrolizumab or nivolumab/ipilimumab or atezolimumab	NA	11.6 10.0
	Enright et al. (2020)	(48)	33 44	NSCLC	SRS + ICI SRS mono	Nivolumab or pembrolizumab or atezolimumab	97 86	13.9
	Shepard et al. (2019)	(49)	34 17	NSCLC	ICI mono SRS + ICI	Nivolumab or pembrolizumab or atezolimumab	76.3 84.9	15.9 NA
	Ahmed et al. (2017)	(52)	17	NSCLC	SRS + ICI	Ipilimumab	NA	5.6
M								
SRS/ICI mono	Rhun et al. (2020)	(53)	10 20 32	M	ICI mono SRS + ST SRS + ICI	Pembrolizumab or nivolumab or ipilimumab	NA	5 13 11
	Trommer et al. (2018)	(54)	26	M	SRS +ICI SRS mono	Pembrolizumab	86 80	NA NA
	Diao et al. (2018)	(55)	40 28 23	M	SRS mono Non-concurrent Concurrent	Ipilimumab	45 70 58	7.8 18.7 11.8
	Kaidar-Person et al. (2017)	(56)	58	M	SRS mono SRS + ICI	Nivolumab or ipilimumab	86 52	5.5 15.0
	Mathew et al. (2013)	(57)	33 25	M	SRS mono SRS + ICI	Ipilimumab	63 65	5.9
	Silk et al. (2013)	(58)	17 16	M	SRS + ICI SRS mono	Ipilimumab	NA NA	19.9 4.0
	Knisely et al. (2012)	(59)	50 11 16	M	SRS mono ICI + SRS SRS + ICI	Ipilimumab	NA NA NA	4.9 19.8 21.3
Concurrent	Rahman et al. (2018)	(60)	35 39	M	Concurrent Non-concurrent	Pembrolizumab or nivolumab or ipilimumab	NA NA	17.8 11.6
	Trommer et al. (2018)	(54)	26	M	SRS +ICI SRS mono	Pembrolizumab	86 80	NA NA
	Diao et al. (2018)	(55)	40 28 23	M	SRS mono Non-concurrent Concurrent	Ipilimumab	45 70 58	7.8 18.7 11.8
	Anderson et al. (2017)	(61)	11	M	Concurrent	Pembrolizumab	NA	NA
	Williams et al. (2017)	(62)	11	M	Concurrent	Ipilimumab	NA	NA
	Yusuf et al. (2017)	(63)	12 6	M	Concurrent Non-concurrent	Pembrolizumab or ipilimumab	87.6 NA	11.9 7.1
	Skrepnik et al. (2017)	(64)	25	M	Concurrent or SRS + ICI	Ipilimumab	94.8	35.8
	Qian et al. (2016)	(65)	33 22	M	Concurrent Non-concurrent	Pembrolizumab or nivolumab or ipilimumab	NA NA	19.1 9.0
	Schoenfeld et al. (2015)	(66)	7 4 5	M	ICI + SRS Concurrent SRS + ICI	Ipilimumab	NA NA NA	6.0 14.4 26.0
	Kiess et al. (2015)	(67)	12 15 19	M	ICI + SRS Concurrent SRS + ICI	Ipilimumab	89 100 87	NA 19.5 NA
ICI ± SRS	Hassel et al. (2022)	(68)	31 19	M	ICI + SRS/WBRT SRS/WBRT + ICI	ipilimumab + nivolumab or ipilimumab	NA	11 15
	Schoenfeld et al. (2015)	(66)	7 4 5	M	ICI + SRS Concurrent SRS + ICI	Ipilimumab	NA NA NA	6.0 14.4 26.0
	Kiess et al. (2015)	(67)	12 15 19	M	ICI + SRS Concurrent SRS + ICI	Ipilimumab	89 100 87	NA 19.5 NA
	Cohen-Inbar et al. (2017)	(69)	14 32	M	ICI + SRS SRS + ICI	Ipilimumab	16.5 54.4	6.4 13.8
	Patel et al. (2017)	(70)	20	M	ICI + SRS	Ipilimumab	71.4	8.0
	Schoenfeld et al. (2015)	(66)	7 4 5	M	ICI + SRS Concurrent SRS + ICI	Ipilimumab	NA NA NA	6.0 14.4 26.0
	Knisely et al. (2012)	(59)	50 11 16	M	SRS mono ICI + SRS SRS + ICI	Ipilimumab	NA NA NA	4.9 19.8 21.3
SRS ± ICI	Hassel et al. (2022)	(68)	31 19	M	ICI + SRS/WBRT SRS/WBRT + ICI	ipilimumab + nivolumab or ipilimumab	NA	11 15
	Rhun et al. (2020)	(53)	10 20 32	M	ICI mono SRS + ST SRS + ICI	Pembrolizumab or nivolumab or ipilimumab	NA	5 13 11
	Carron et al. (2020)	(71)	50	M	SRS + ICI	Pembrolizumab or nivolumab	94	16.6
	Galli et al. (2019)	(72)	18 18	M	WBRT + ICI SRS + ICI	Pembrolizumab or nivolumab or ipilimumab	NA NA	5.0 7.0
	Murphy et al. (2019)	(73)	26	M	SRS + ICI	Pembrolizumab or nivolumab or ipilimumab	NA	26.1
	Minniti et al. (2019)	(74)	35 45	M	SRS + ICI SRS + ICI	Ipilimumab Nivolumab	70 85	14.7 22.0
	Robin et al. (2018)	(75)	38	M	SRS + ICI	Nivolumab or ipilimumab	NA	NA
	Nardin et al. (2018)	(76)	25	M	SRS + ICI	Pembrolizumab	80	15.3
	Trommer et al. (2018)	(54)	26	M	SRS +ICI SRS mono	Pembrolizumab	86 80	NA NA
	Kaidar-Person et al. (2017)	(56)	58	M	SRS mono SRS + ICI	Nivolumab or ipilimumab	86 52	5.5 15.0
	Skrepnik et al. (2017)	(64)	25	M	Concurrent or SRS + ICI	Ipilimumab	94.8	35.8
	Cohen-Inbar et al. (2017)	(69)	14 32	M	ICI + SRS SRS + ICI	Ipilimumab	16.5 54.4	6.4 13.8
	Choong et al. (2017)	(77)	108	M	SRS + ICI	NA	78	14.2
	Ahmed et al. (2016)	(52)	26	M	SRS + ICI	Ipilimumab	82	12.0
	Schoenfeld et al. (2015)	(66)	7 4 5	M	ICI + SRS Concurrent SRS + ICI	Ipilimumab	NA NA NA	6.0 14.4 26.0
	Kiess et al. (2015)	(67)	12 15 19	M	ICI + SRS Concurrent SRS + ICI	Ipilimumab	89 100 87	NA 19.5 NA
	Tazi et al. (2015)	(78)	10	M	SRS + ICI	Ipilimumab	NA	29.3
	Mathew et al. (2013)	(57)	33 25	M	SRS mono SRS + ICI	Ipilimumab	63 65	5.9
	Silk et al. (2013)	(58)	17 16	M	SRS + ICI SRS mono	Ipilimumab	NA NA	19.9 4.0
	Knisely et al. (2012)	(59)	50 11 16	M	SRS mono ICI + SRS SRS + ICI	Ipilimumab	NA NA NA	4.9 19.8 21.3
M, NSCLC, RCC, and others								
SRS/ICI mono	Kowalski et al. (2020)	(79)	179	NSCLC, M, RCC	SRS + ICI SRS mono	Durvalumab or atezolimumab or pembrolizumab or nivolumab or ipilimumab	98.0 89.5	NA
	Lanier et al. (2019)	(80)	101 170	NSCLC, M, other	SRS + ICI SRS mono	Ipilimumab or nivolumab/ipilimumab or pembrolizumab or nivolumab	91 96	15.9 6.1
	Chen et al. (2018)	(81)	181 28 51	NSCLC, M, RCC	SRS mono Concurrent Non-concurrent	Pembrolizumab or nivolumab or ipilimumab	82 88 79	12.9 24.7 14.5
Concurrent	Trommer et al. (2022)	(82)	41 SRS + 22 WBRT 24 SRS + 6 WBRT	NSCLC, M, other	Concurrent Non-concurrent	Pembrolizumab or nivolumab	95.3 69.2	17.6 6.8
	Travis et al. (2021)	(83)	110	NSCLC, M	Concurrent Non-concurrent	Nivolumab and/or pembrolizumab or Ipilimumab	NA	14.2
	Koenig et al. (2019)	(84)	97	NSCLC, M, RCC, other	Concurrent Non-concurrent	Pembrolizumab or nivolumab or ipilimumab	96 97	9.4
	Chen et al. (2018)	(81)	181 28 51	NSCLC, M, RCC	SRS mono Concurrent Non-concurrent	Pembrolizumab or nivolumab or ipilimumab	82 88 79	12.9 24.7 14.5
SRS ± ICI	Qian et al. (2020)	(85)	74	NSCLC, M, RCC	SRS + ICI	Durvalumab or pembrolizumab or ipilimumab	90.3	NA
	Kowalski et al. (2020)	(79)	179	NSCLC, M, RCC	SRS + ICI SRS mono	Durvalumab or atezolimumab or pembrolizumab or nivolumab or ipilimumab	98.0 89.5	NA
	Lanier et al. (2019)	(80)	101 170	NSCLC, M, other	SRS + ICI SRS mono	Ipilimumab or nivolumab/ipilimumab or pembrolizumab or nivolumab	91 96	15.9 6.1

#, number; ICI, immune checkpoint inhibitor; LTC, local tumor control; OS, overall survival; NSCLC, non-small cell lung cancer; M, melanoma; RCC, renal cell carcinoma; mono, monotherapy; SRS, stereotactic radiosurgery; CT, chemotherapy; NA, not applicable; mo, months.

**Table 3:** Completed or ongoing clinical trials of stereotactic radiosurgery and immune checkpoint inhibitors in brain metastases treatment.

Clinical Trial	Phase	Tumor	ICI Target/Drug	Modalities
NCT01703507	1	M	CTLA-4/Ipilimumab	WBRT vs. SRS
NCT01950195	1	M	CTLA-4/Ipilimumab	NA
NCT02107755	2	M	CTLA-4/Ipilimumab	NA
NCT02696993	2	NSCLC	CTLA-4/Ipilimumab, PD-1/Nivolumab	WBRT vs. SRS
NCT02716948	1	M	PD-1/Nivolumab	NA
NCT02858869	1	NSCLC, M	PD-1/Pembrolizumab	SRS
NCT02886585	2	M	PD-1/Pembrolizumab	NA
NCT02978404	2	NSCLC, RCC	PD-1/Nivolumab	NA
NCT03340129	2	M	CTLA-4/Ipilimumab, PD-1/Nivolumab	SRS
NCT03807765	1	BC	PD-1/Nivolumab	NA
NCT04047602	1	NSCLC, M, others	NA	SRS
NCT04427228 (MIGRAINE)	2	NSCLC, M, others	NA	SRS
NCT04650490 (STICK-IM)	2	NSCLC	NA	NA
NCT04889066	2	NSCLC	PD-L1/Durvalumab	fSRT or PULSAR
NCT04711824	2	BC	PD-L1/Durvalumab	RT

ICI, immune checkpoint inhibitor; LTC, local tumor control; OS, overall survival; NSCLC, non-small cell lung cancer; M, melanoma; RCC, renal cell carcinoma; BC, breast cancer; mono, monotherapy; SRS, stereotactic radiosurgery; WBRT, whole-brain radiation therapy; PULSAR, ersonalized ultra-fractionated stereotactic adaptive radiotherapy; NA, not applicable.

## Clinical evidence of combination therapy

Ionizing radiation is known to cause DNA damage and subsequent cell death and its impact on the immune system has been extensively studied in recent years (86). Local RT can stimulate an antitumoral immune response by releasing TAAs from necrotic and apoptotic tumor cells, which are presented to CD8+ cytotoxic T cells by DCs (87). This activates the immune system to attack tumor cells throughout the body (88). Numerous preclinical and clinical studies have demonstrated that combining RT with ICIs can significantly enhance the systemic immune response, resulting in immunogenic tumor cell death. This approach has shown promising results in improving treatment outcomes and may be a valuable strategy for cancer management.

## Clinical standpoint

From a clinical perspective, the application of SRS and ICIs for the treatment of brain metastasis has garnered significant interest, driven by preclinical and theoretical evidence. A number of studies have examined the optimal treatment sequence, SRS fractionation regimen and dosing, appropriate selection of ICIs, therapeutic efficacy, and potential adverse effects, generating a debate among experts in the field. The complexities surrounding optimal treatment strategies for brain metastasis are further compounded by the reliance on retrospective cohort analyses with small sample sizes, despite reporting improved LTC and OS rates with acceptable toxicity profiles. Notably, these studies primarily involve patients with M and the utilization of ipilimumab as the most frequently administered ICI. To address the current challenges, we present a comprehensive overview of studies examining brain metastasis in various cancer types, including NSCLC (47–52), M (53–78, 89, 90), and RCC among others (68, 79, 80, 82–85) (**Table 2**).

The definition of concomitant administration of ICI and SRS exhibits significant variability among studies. A notable portion of the literature defines concomitance as the simultaneous delivery of SRS and ICI within a timeframe of four weeks before or after the initiation of ICI (55, 69, 71, 73, 84, 91). Although some studies employ a narrower definition of concomitance with a window of less than two weeks (74, 81), others define it as extending up to over 2 months (61, 92).

Numerous studies have conducted comparisons between exclusive SRS monotherapy and combined ICI-SRS treatment with the overall consensus indicating improved LTC rates and patient outcomes with the concomitant administration of SRS and ICI (54, 93). Moreover, the combined SRS-ICI treatment resulted in a significant decrease in local failure compared to SRS monotherapy in M brain metastases (94). In addition, the combination of ICI and SRS may also provide benefit for PFS compared to SRS alone (55, 91).

Furthermore, a trend towards improved patient outcomes and LTC rates was observed in the patients who received concomitant SRS-ICI treatment, compared to those who received sequential treatment with no discernible difference in toxicity (81). The impact of combining SRS with ICI on treatment-related toxicity remains a subject of debate. While some studies have reported grade 3 or higher toxicity rates ranging from 5% to 24%, other investigations have identified a higher incidence of symptomatic radionecrosis after SRS-ICI treatment (hazard ratio 2.56, 95% confidence interval: 1.35–4.86, p = 0.004). Therefore, the true extent of toxicity resulting from this treatment modality remains inconclusive (95).

To provide a more comprehensive evaluation of the efficacy and safety of the combination of SRS and ICIs for brain metastasis, meta-analyses are warranted, given the limited patient population and the diversity of SRS and ICI treatment regimens across studies (96). One such meta-analysis found that concurrent administration of ICIs and SRS led to a statistically significant improvement in 1-year OS compared to non-concurrent ICI administration (96, 97).

While previous studies have yielded valuable insights into the combination of SRS and ICIs, it is imperative to recognize that their conclusions are based on retrospective observational studies, which may not fully reflect the extent of their effects. The true therapeutic value of this modality can only be established through well-designed prospective clinical trials, which aim to minimize biases and confounding variables that may influence the results of retrospective observational studies. Therefore, it is crucial to await the results of these trials to gain a more robust understanding of the impact of combining SRS and ICIs on patient outcomes and to provide healthcare professionals with a clear guidance to make informed treatment decisions.

## Optimal dose and fractionation

Achieving the optimal dose and fractionation of radiation in SRS for treating spontaneous brain metastases poses a complex clinical challenge due to the need to balance effective tumor cell cytotoxicity against suppression of radiosensitive lymphocyte (98). Advancements in technology have enabled delivery of high radiation doses in a fractionated manner over multiple days or as a single fraction, thereby addressing this challenge (98). Preclinical studies provide limited insight into the complex interactions that occur within the CNS, but still furnish valuable information. For instance, in a murine M model, single-fraction doses ranging from 7.5 to 15 Gy optimized LTC, with a dose of 7.5 Gy providing a balance between tumor control and minimal suppression of Tregs. Fractionating the 15 Gy dose into two or three smaller doses improved LTC, reduced Tregs, and elicited an immune response (39).

The appropriate timing for administering immunotherapy in conjunction with RT remains a topic of ongoing research. Administering immunotherapy prior to SRS could enhance the antitumor response by allowing APCs and effector cells to be present when tumor cells are destroyed. However, this sequence could result in a reduced response if the circulating lymphocytes are recruited and then damaged by subsequent radiation. On the other hand, administering RT prior to immunotherapy could enhance expression of TAAs and increase BBB permeability, potentially improving drug delivery and immune cell infiltration. The optimal sequence of immunotherapy and RT depends on various factors, including radiation delivery parameters, the mode of action of immunotherapy, tumor histology, and overall mutational profile (99).

It is widely accepted that ICI therapy should be administered either in conjunction with or prior to SRS or RT. A preclinical study of colorectal cancer treatment with a combination of 2 Gy x 5 fractions of radiation and a PD-L1 inhibitor has demonstrated that simultaneous administration of the inhibitor on either the first or fifth day is the most efficacious approach. However, administering the inhibitor seven days after radiation completion failed to improve survival compared to RT alone (100). The optimal scheduling of SRS or RT in combination with immunotherapy remains a topic of ongoing investigation and research.

In a series of experiments evaluating the effect of the timing of administration of anti-CTLA-4 and anti-OX40 antibodies in relation to a single dose of 20 Gy radiation, the optimal timing of anti-CTLA-4 administration in relation to RT was found to depend on the dose and timing of the antibody (101). Administering anti-CTLA-4 prior to RT cleared all tumors, whereas administering it after RT resulted in only 50% of tumors being eliminated. The optimal timing of administering anti-OX40 was only after radiation completion, and mice that received anti-OX40 1 day after radiation showed improved tumor clearance and a doubling of median survival time. These results suggest that anti-CTLA-4 therapy in combination with RT improves outcomes, regardless of when the therapy is administered (101).

The order in which anti-CTLA-4 antibodies are administered has a significant effect on treatment efficacy. Prior studies have indicated that administering the drug either two days before or concurrent with radiation completion leads to improved treatment outcomes compared to administration two days after radiation (102). Administering the CTLA-4 inhibitor ipilimumab during or after SRS has demonstrated better survival rates compared to pretreatment administration in clinical practices. This outcome may occur because radiation releases antigens and prepares the immune system prior to the administration of ipilimumab (67). Optimal results were obtained when both local and systemic modalities were delivered within a four-week window for patients with M brain metastases who underwent SRS and received CTLA-4 or PD-1 inhibitors (65).

## Conclusion

Recent evidence highlights a paradigm shift in our knowledge of the BBB and its role in brain metastases treatment. While traditionally considered a protective barrier against immune cells, emerging data suggests that certain immune cells and treatments can penetrate the BBB, opening new avenues for therapeutic interventions. As a result, the combination of SRS and immunotherapy has gained significant interest as a potential synergistic approach to combat brain tumors.

Radiation has been found to enhance the immune response, and T-cell-mediated responses are crucial in controlling tumors post-radiation locally and systemically. The potential benefit of combining of SRS and ICIs are promising, including improved OS, reduced local failure rates, and decreased risk of local recurrence, surpassing the outcomes of either treatment as a monotherapy. However, it is essential to acknowledge that existing data on this combination primarily stems from single-center retrospective cohort studies, warranting further investigation.

The optimal administration sequence of immunotherapy with SRS remains uncertain and may vary depending on the specific systemic immunotherapy agent utilized. As we delve deeper into intricacies of this treatment combination, the possibility of increased incidence of severe adverse events compared to monotherapies still requires definitive determination.

In light of these advancements, we need to consider whether SRS might lose its necessity in the treatment of brain metastases due to the efficacy of immunotherapy. This question challenges us to identify predictive factors that enable better patient selection for initial immunotherapy, while reserving SRS for cases of intracranial progression. Recognizing the importance of patient selection, we must develop strategies to tailor treatment plans for individual cases, optimizing therapeutic outcomes and reducing treatment-related toxicities. For instance, recent findings supporting the efficacy of immunotherapy in treating brain metastases underscores its clinical relevance (103). Nonetheless, it is crucial to remain cautious and focused on refining our treatment approaches to strike a delicate balance between efficacy and safety.

In conclusion, the synergistic integration of SRS and immunotherapy holds immense potential in revolutionizing brain metastases treatment. To fully harness the potential of this combination, there is an imperative need to conduct additional well-designed prospective studies that elucidate the intricate interplay between SRS and ICIs. These studies hold the key to establishing robust clinical guidelines and tailored treatment plans, optimizing therapeutic outcomes for patients while mitigating the risk of treatment-related toxicities.

As we pursue advancement in this dynamic field, we aspire to propel the frontiers of neuro-oncology in patients facing the challenges of brain metastases.

## Author contributions

DP and KY have made a substantial contribution to the concept and design of the article. KY drafted the article. SC and DP revised it critically for important intellectual content. ML approved the version to be published. All authors agreed to be accountable for all aspects of the work in ensuring that questions related to the accuracy or integrity of any part of the work are appropriately investigated and resolved.
